# Surfactant-Free Monodispersed Pd Nanoparticles Template for Core-Shell Pd@PdPt Nanoparticles as Electrocatalyst towards Methanol Oxidation Reaction (MOR)

**DOI:** 10.3390/nano12020260

**Published:** 2022-01-14

**Authors:** Fulin Zheng, Tsz-Lung Kwong, Ka-Fu Yung

**Affiliations:** Department of Applied Biology and Chemical Technology, The Hong Kong Polytechnic University, Hung Hom, Kowloon, Hong Kong; 09902085@connect.polyu.edu.hk

**Keywords:** core-shell, PdPt alloy, nanoparticles, electrocatalysis, fuel cells, methanol oxidation

## Abstract

An eco-friendly two-step synthetic method for synthesizing Pd@PdPt/CNTs nanoparticles was introduced and studied for the methanol oxidation reaction. The Pd@PdPt alloy core-shell structure was synthesized by preparing a surfactant-free monodispersed Pd/CNTs precursor through the hydrolysis of tetrachloropalladate (II) ion ([PdCl_4_]^2−^) in the presence of carbon nanotubes (CNTs) and the subsequent hydrogen reduction and followed by a galvanic replacement reaction. This method opens up an eco-friendly, practical, and straightforward route for synthesizing monometallic or bimetallic nanoparticles with a clean surfactant-free electrocatalytic surface. It is quite promising for large-scale preparation. The Pd@PdPt/CNTs electrocatalyst demonstrated a high specific mass activity for methanol oxidation (400.2 mAmg_Pt_^−1^) and excellent stability towards direct methanol oxidation compared to its monometallic counterparts.

## 1. Introduction

Critical issues such as global warming, the energy crisis, and air pollution have stimulated intensive research on energy storage and conversion from alternative energy sources [1,2,3,4,5]. Direct methanol fuel cells (DMFCs) are attractive energy conversion devices that have received extensive studies in the last couple of decades [6,7,8,9,10,11,12]. Compared to proton exchange membrane fuel cells (PEMFCs), DMFCs exhibit a unique advantage because methanol is an abundant and inexpensive liquid fuel with a high volumetric energy density and easy handling, storing, and transporting [13,14].

Due to the excellent performance activity and durability, platinum (Pt)-based nanomaterials are the most promising electrocatalysts used as the cathode and anode in DMFCs [15,16,17,18]. However, a large amount of Pt is required for a practical activity with high costs that hinders the commercialization of PEMFCs. In the literature, researchers have been devoted to developing electrocatalysts with improved Pt use and enhanced catalytic activity [19,20,21,22]. Among them, palladium-platinum (PdPt) electrocatalysts have received considerable interest recently as Pd costs much less than Pt (one-quarter of Pt). Additionally, the electrochemical stability of Pd is higher than other transition metals such as iron (Fe), copper (Cu), nickel (Ni), or cobalt (Co). Co-reduction is one of the most commonly used and convenient approaches to prepare PdPt electrocatalysts [23,24,25,26]. The enhanced electrocatalytic activity is mainly due to the electronic effect caused by the interaction between Pd and Pt. This synergetic effect correlated with the composition of PdPt electrocatalyst was also reported though there was no consensus concerning the optimal molar ratio due to variations in the experimental conditions [26,27,28,29]. A template-assisted method is versatile for synthesizing PdPt materials with various morphologies since the final products usually inherit the same morphology as the templates [30,31,32]. The morphology of the PdPt was proved to greatly influence the electrocatalytic activity of the catalysts originating from the increase in surface area and the changes in surface crystallinity. PdPt-based core-shell electrocatalysts have recently received considerable attention owing to their excellent electrochemical performance. Pd@Pt core-shell structure can be achieved by electrodeposition [33], galvanic displacement [12,34,35], or physical process [36,37]. The coating of Pt on the Pd surface will greatly increase the surface area of Pt, thus enhancing Pt use. Moreover, the core and shell interaction gives electrocatalytic activity and improved stability. All the methods mentioned above involve surfactants to achieve a small size of better size distribution.

Herein, green surfactant-free synthesis of Pd@PdPt/CNTs nanocomposite via a two-step method for highly efficient methanol electro-oxidation has been investigated. The surfactant-free synthetic process can even control the size and distribution of Pd nanoparticles on the carbon nanotubes (CNTs). Pd/CNTs template precursor was first prepared through a green surfactant-free synthetic method involving the hydrolysis of [PdCl_4_]^2−^ to yield monodispersed palladium oxide (PdO), which were then reduced by hydrogen to produce Pd nanoparticles. This green synthetic method provides a surfactant-free (i.e., clean) Pd surface to construct Pd@PdPt structure via galvanic replacement and no further washing step is required to remove surfactants and capping agents. This method is beneficial for the large-scale production of this kind of electrocatalyst. Due to the non-involvement of surfactants and capping agents in the synthetic process, the expression of an absolutely clean and active electrocatalytic surface benefits galvanic replacement reaction and electro-oxidation of methanol. Electrochemical studies demonstrated that the as-prepared Pd@PdPt/CNTs displayed superior performance for methanol oxidation.

## 2. Materials and Methods

### 2.1. Synthesis of Electrocatalyst

#### 2.1.1. Preparation of Pd/CNTs Template Composite Precursor

The multi-wall carbon nanotubes (MWCNTs) were purchased from Fortune. MWCNTs (100 mg) was refluxed in concentrated nitric acid (HNO_3_, 65 wt.%, 100 mL) under 80 °C for 16 h. The black acid-treated MWCNTs was centrifuged and washed until a pH value of around 6 was achieved. The acid-treated MWCNTs was dried under 80 °C for 16 h and kept for further use.

Acid-treated multi-wall carbon nanotubes (MWCNTs, 0.2 mg) were dispersed in Milli-Q water (50 mL) under ultrasonic for 30 min. The black dispersion was heated to 60 °C at an oil bath. A desired amount of K_2_PdCl_4_ was added into the above dispersion while stirring. The mixture was then stirred for 2 h to complete hydrolysis [PdCl_4_]^2−^. The black solid was separated by centrifugation and washed twice with Milli-Q water.

For the reduction process, the as-obtained black solid was re-dispersed in Milli-Q water (50 mL) in a round-bottom flask and was bubbled with pure hydrogen for 10 min. The flask was sealed under hydrogen protection and the mixture was heated to 60 °C for 2 h. The product was collected by centrifugation, washed, and dried. The preparation process for the Pt/CNTs was the same as that of the Pd/CNTs composite precursor.

#### 2.1.2. Preparation of Pd@PdPt/CNTs

Pd/CNTs template composite precursor (1 mg) was dispersed into Milli-Q water (5 mL) in a vial by sonication for 5 min. A desired amount of K_2_PtCl_4_ was added to the dispersion. The vial was heated to 90 °C and stirred for 1 h. The product was collected by centrifugation, washed with Milli-Q water, and dried.

### 2.2. Materials Characterization

The morphologies of the as-synthesized materials were characterized by a transmission electron microscope (TEM) equipped with energy-dispersive X-ray spectroscopy (EDX). High-resolution transmission electron microscope (HR-TEM) and selected area electron diffraction (SAED) were performed on the field emission-transmission electron microscope (FE-TEM, JEOL JEM-2011F).

The crystal planes of the as-synthesized materials were identified by powder X-ray diffraction (XRD) using Rigaku SmartLab with a CuKα (λ = 1.541862 Å) radiation, operating at 45 kV and 200 mA, with a scanning rate of 0.05°/s and 2-theta ranging from 20° to 90°.

X-ray photoelectron spectroscopy (XPS) studies were also carried out using Axis Ultra DLD XPS system equipped with monochromatic Al−Kα radiation of 1486.6 eV and an electron take-off angle of 90°. The pressure of the sample chamber was kept at 10^−8^ Torr during analysis. The spectrum was recorded in the binmr1ding energy (B.E.) range of 0.00 to 1400.00 eV with a step size of 1.00 eV. The binding energy was referenced with the C 1s peak of the carbon at 285.0 eV.

The mass loading on the carbon nanotubes supporting samples was measured by induced coupled plasma mass spectrometry (ICP-MS).

### 2.3. Electrochemical Measurements

All electrochemical measurements were conducted on the CHI660D electrochemistry station at room temperature using a conventional three-electrode system. The saturated calomel electrode (SCE) and Pt foil were used as the reference and counter-electrodes, respectively. All the potentials reported referred to SCE unless otherwise stated. For the preparation of the working electrode, the electrocatalyst (1 mg) was dispersed into a mixture of water (800 µL) and isopropanol (200 µL) under ultrasonic for 30 min. The dispersion solution (2 µL) was cast on the glassy carbon electrode (GCE). After drying in air, Nafion solution (0.05%, 2 µL) was dropped on the GCE to improve the attachment. Cyclic voltammetry and chronoamperometry were performed for diagnostic purposes and catalytic activity investigation. The working electrolyte was purged with pure nitrogen for 15 min before each measurement.

## 3. Results and Discussions

### 3.1. Synthesis of Pd@PdPt/CNTs Electrocatalyst

**Scheme 1:**
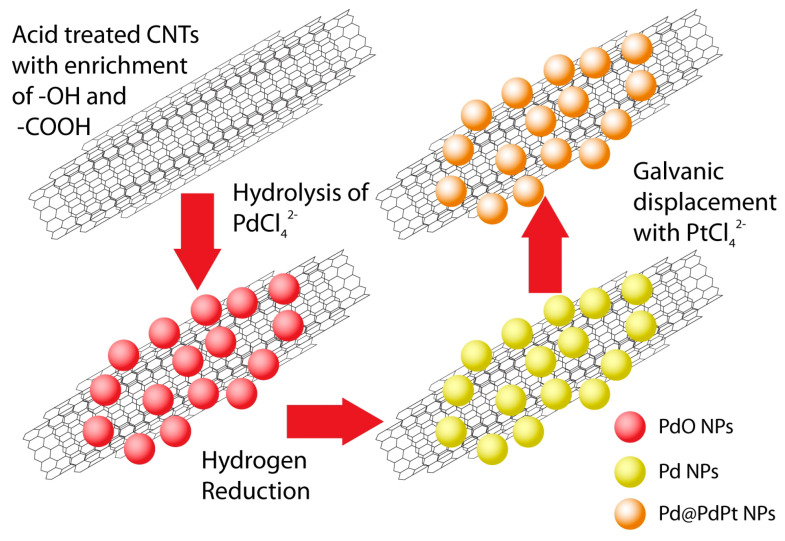
The schematic diagram illustrates the synthesis of Pd@PdPt/CNTs nanoparticle electrocatalyst.

The synthetic diagram of Pd@PdPt/CNTs is illustrated in Scheme 1. PdO/CNTs was synthesized by hydrolysis of K_2_PdCl_4_ in the presence of carbon nanotubes (CNTs). Due to the high affinity of Pd toward oxygen, [PdCl_4_]^2−^ would first coordinate with the oxygen-containing functional groups present on the CNTs surface and slowly hydrolyze to yield supported monodispersed PdO nanoparticles. The resulting PdO was subsequently reduced to Pd nanoparticles by hydrogen under 60 °C to generate a surfactant-free surface for further modification. PdPt alloy coating of the Pd nanoparticles was achieved by the galvanic replacement between [PtCl_4_]^2−^ and Pd. Several reports in the literature discussed that the driving force for alloy formation is mainly due to the difference in the surface free energy [38,39]. The surface free energies of Pt and Pd are 2.204 Jm^−2^ and 1.743 Jm^−2^ respectively [40]. Based on this premise, the significant difference of surface free energy makes the direct PdPt alloying process occur on the surface of Pd nanoparticles.

This synthetic protocol is environmentally benign as it does not involve any surfactants and capping agents. It can achieve better particle size distribution and avoid further washing steps to remove surfactants. Hence a clean electrocatalytic surface is expressed, which is beneficial and ensures efficient galvanic replacement and the later electro-oxidation of methanol. Moreover, it is applicable for mass production with a high demand for fuel cell electrocatalysts production.

### 3.2. Physical Characterization of Electrocatalysts

CNT is a superior supporting material for precious metals such as Pt and Pd to enhance their use when used as electrocatalysts for PEMFCs. It has stimulated numerous research works in studying the electrochemistry of CNTs supported metal nanoparticles [41,42,43]. It has been reported recently that CNTs could be used as supporting material and reducing agents for the fabrication of M/CNTs composites (where M is Pt or Au) [44]. Xie et al. also demonstrated successful synthesis of Pd/graphene composite through the redox reaction between [PdCl_4_]^2−^ and graphene.

**Figure 1:**
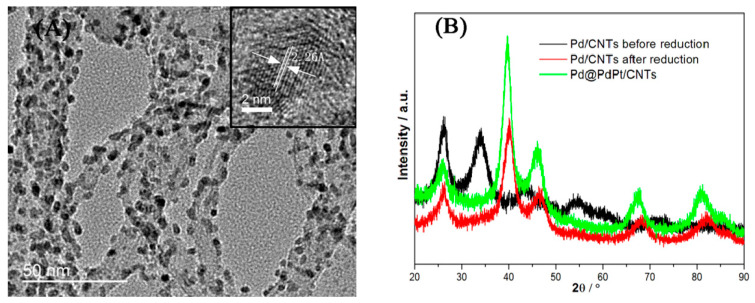

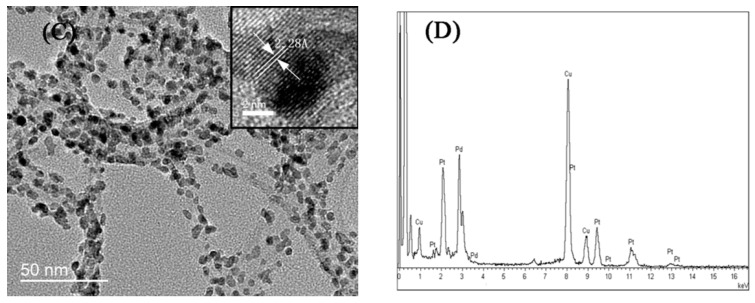
(**A**) TEM image of Pd/CNTs composite (insets: HR-TEM), (**B**) XRD patterns of PdO/CNTs, Pd/CNTs and Pd@PdPt/CNTs, (**C**) TEM image of Pd@PdPt/CNTs composite (insets: HR-TEM), and (**D**) EDX pattern of Pd@PdPt/CNTs.

However, in our present work, the PdO/CNTs composite was obtained before the reduction by hydrogen, which was examined by XRD analysis (Figure 1b, labeled in black). A relatively strong diffraction peak appears with a diffraction angle of 34 degrees in the diffraction pattern corresponding to the [101] crystal plane of PdO [45]. The XRD pattern of as-synthesized PdO/CNTs aligns well with the standard diffraction pattern, supporting our proposal of oxide formation.

The discrepancy between our synthetic protocol and that reported in the literature might be the different synthetic reaction conditions. A relatively high temperature (60 °C) in our presented preparation was employed, whereas 0 °C was chosen in Xie’s preparation. Hydrolysis of [PdCl_4_]^2−^ could occur at 60 °C to form stable PdO nanoparticles, hindering the electron transfer from CNTs to Pd^2+^. Moreover, the redox potential of graphene and CNTs also plays a critical role as it closely depends on the physical state of the graphene layer [46,47].

Hydrogen was chosen as the reducing agent to reduce PdO to Pd as it does not contaminate any metal particles with organic residue. The formation of Pd nanoparticles was proven by the XRD analysis, which shows typical face center cubic (fcc) diffractions (Figure 1b, labeled in red) with the most intensive diffraction peak of [111] crystal plane. The morphology of the Pd/CNTs composite was characterized by TEM. As shown in Figure 1a, monodispersed Pd nanoparticles with a diameter of 3.5 nm are decorated on the surface of CNTs. HR-TEM and FFT confirm that the nanoparticles present are Pd with a d-spacing of 2.26 Å, which is corresponded to the [111] crystal plane of Pd.

**Figure 2:**
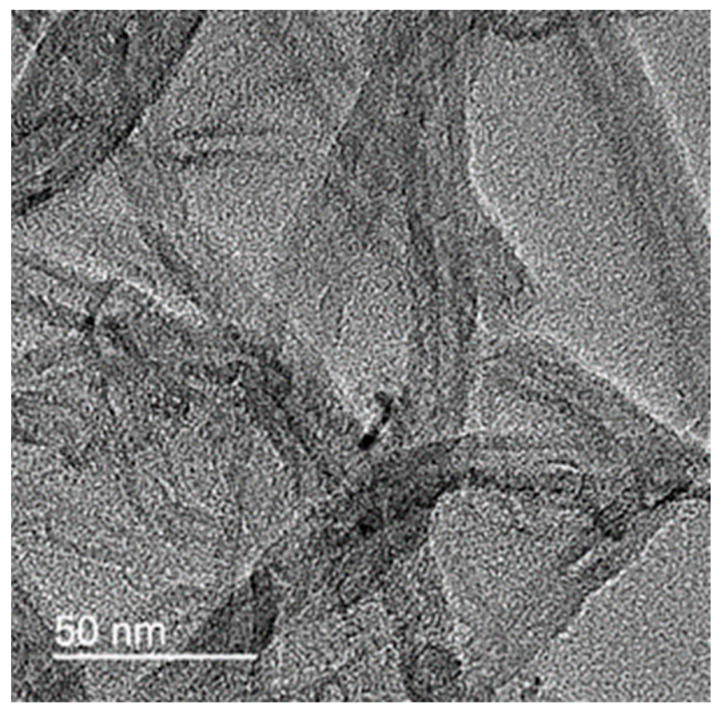
TEM image of the sample prepared under H_2_SO_4_ (0.1 M).

To further confirm the formation of PdO, the same procedure was repeated in H_2_SO_4_ (0.1 M) instead of Milli-Q water. The TEM micrograph of the resultant CNTs confirms that no nanoparticles are formed on its surface (Figure 2). It suggests that the hydrolysis [PdCl_4_]^2−^ is wholly inhibited under an acidic medium.

**Figure 3:**
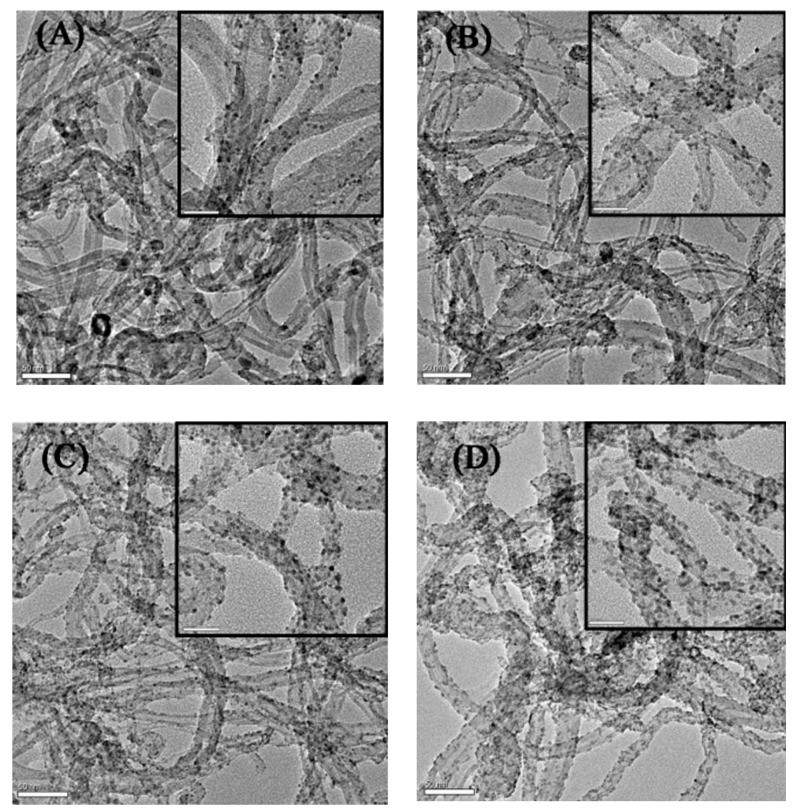

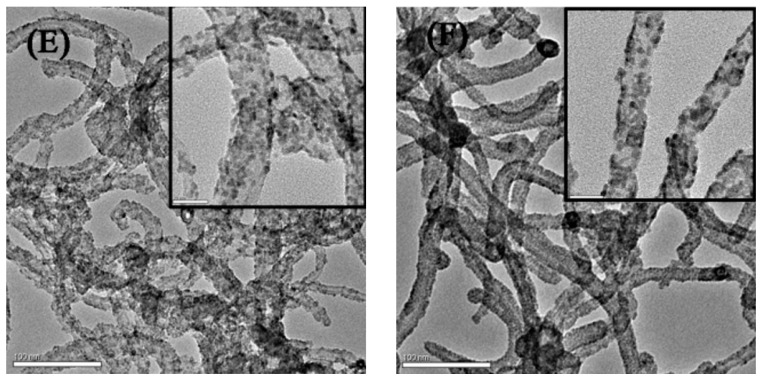
TEM images of Pd/CNTs composite with different metal loading (**A**) 6.1%, (**B**) 11.5%, (**C**) 20.6%, (**D**) 33.8%, (**E**) 48.6%, and (**F**) 58.0%.

Furthermore, the introduction of [PdCl_4_]^2−^ to CNTs led to a decrease in the solution pH by 2 (Table 1), which implies that hydrolysis takes place. To investigate the effect of the Pd precursor on the overall loading, the hydrolysis was repeated using solutions with different concentrations of Pd ions. Increasing the concentration of the initial [PdCl_4_]^2−^ solution, the overall metal loading increased from 6.1% to 58.0%. The sizes of the Pd nanoparticles were also increased slightly from 2.3 nm to 3.6 nm (Figure 3). Despite increasing the overall Pd loading, no significant nanoparticle aggregation is observed even without surfactants and capping agents.

The as-prepared surfactant-free Pd/CNTs was allowed to be stirred in [PtCl_4_]^2−^ solution for the galvanic replacement to yield Pd@PdPt/CNTs. After decoration by Pt, no apparent changes in morphology are observed, as shown in Figure 1c. However, a d-spacing of 2.28 Å correlating with the [111] crystal plane is obtained by the HR-TEM (inset of Figure 1c), indicating the formation of a PdPt alloy [31,32,33,48]. The slight increase in d-spacing was further confirmed by XRD measurement in which a slight decrease in all diffractions peaks is observed. Furthermore, the EDX analysis also proves the presence of the Pt with the atomic ratio of Pd to Pt of 2.6:1 as shown in Figure 1d.

### 3.3. Electrochemical Characterization of Pd@PdPt/CNTs Electrocatalyst

**Figure 4:**
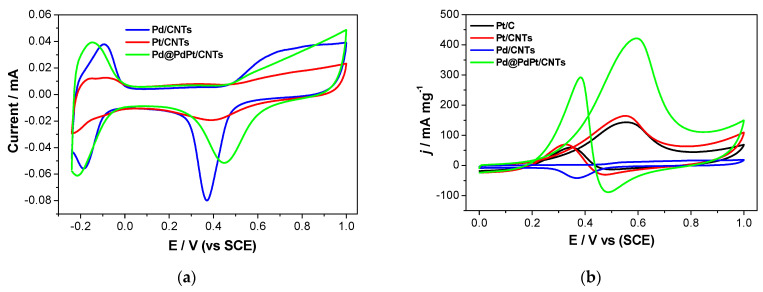
(**a**) CVs of different electrocatalysts in 0.1 M H_2_SO_4_, 50 mVs^−1^ and (**b**) CVs of different electrocatalysts in 0.1 M H_2_SO_4_ + 0.1 M methanol. Current was normalized to the mass of metals, 50 mVs^−1^.

The as-prepared composite was characterized by cyclic voltammetry in H_2_SO_4_ (0.1 M) to further verify the successful decoration of Pd surface by PdPt alloying. The CV curves for Pd/CNTs, Pt/CNTs and Pd@PdPt/CNTs are presented in Figure 4a. A noticeable change can be observed for the Pd/CNTs after modification by Pt. In the characteristic hydrogen region, namely between −0.24 V and 0 V (vs SCE), both the adsorption and desorption peaks shifted negatively. On the other hand, considering the metal oxide reduction region between 0.4 V and 0.8 V, the intensity of the metal oxide reduction peak decreased for Pd@PdPt/CNTs compared to that of Pd/CNTs accompanied by a positively shift of the onset potential, which indicates the coating of PdPt alloy on the surface of Pd nanoparticles. These results can be attributed to the presence of PdPt alloy on the surface of Pd nanoparticles, which could modify the hydrogen adsorption and desorption characteristics and the metal oxide formation and reduction upon electrochemical cycling [49].

**Figure 5:**
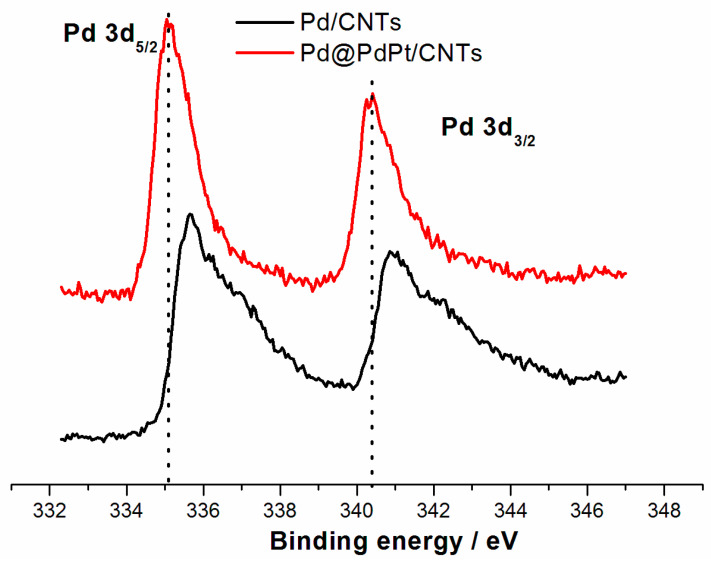
XPS spectra for the Pd 3d core levels of Pd/CNTs and Pd@PdPt/CNTs.

The Pd@PdPt/CNTs catalyst displayed an excellent electrochemical performance towards methanol oxidation. The CVs of methanol oxidation in H_2_SO_4_ solution (0.1 M) containing methanol (0.1 M) using Pt/C, Pt/CNTs Pd/CNTs, and Pd@PdPt/CNTs are displayed in Figure 4b. The current values were normalized to the total metal mass of catalysts measured by ICP-MS. It is well known that Pd is inert towards methanol oxidation. The CV of Pd/CNTs in H_2_SO_4_ (0.1 M) containing methanol (0.1 M) shows no observable peak corresponding to methanol oxidation which confirms the inactiveness of Pd towards methanol oxidation. However, the Pd@PdPt/CNTs displayed superior activity for methanol, as demonstrated by the CVs in Figure 4. The specific mass activity at 0.55 V on Pd@PdPt/CNTs (400.2 mAmg_Pt_^−1^) is 2.8 times that of commercially available Pt/C (142.8 mAmg_Pt_^−1^) and 2.4 times of Pt/CNTs (164.1 mAmg_Pt_^−1^). Compared to Pt containing electrocatalyst towards methanol oxidation reaction in the literature, as-synthesized Pd@PdPt/CNTs demonstrates a higher specific mass activity as summarized in Table 2. Moreover, the onset potential for the methanol oxidation revealed a negative shift of around 40 mV for the Pd@PdPt/CNTs (ca. 0.117 V) compared to that of Pt/C and Pt/CNTs (ca. 0.153 V). The enhanced electrocatalytic activity of the Pd@PdPt/CNTs could be ascribed to the synergetic effect caused by the formation of the PdPt alloy. The PdPt alloy has a modified electronic structure compared to its monometallic counterparts; therefore, the improvement of electrocatalytic properties results [50,51,52]. To further investigate the electronic structure of electrocatalysts, X-ray photoelectron spectroscopy (XPS) was conducted to study the binding energy of the Pd atoms present. As depicted in Figure 5, the binding energy of Pd_5/2_ and Pd_3/2_ for Pd@PdPt/CNTs and Pd/CNTs was 335.1 eV, 340.4 eV and 335.7 eV, 340.9 eV, respectively. The binding energy for the Pd@PdPt/CNTs was lower than that of the Pd/CNTs unambiguously, indicating the modification of the electronic structure due to the alloy formation. The surface ratio of Pd to Pt measured by XPS analysis is found to be around 1:1, which is lower than the atomic ratio of Pd to Pt measured by EDX analysis (2.6:1). It could be explained that XPS analysis is a surface characterization method without detecting the signal originating from the Pd core and thus resulting in a higher atomic ratio of Pt. This finding is consistent with the Pd@PdPt/CNTs core-shell structure as proposed.

**Figure 6:**
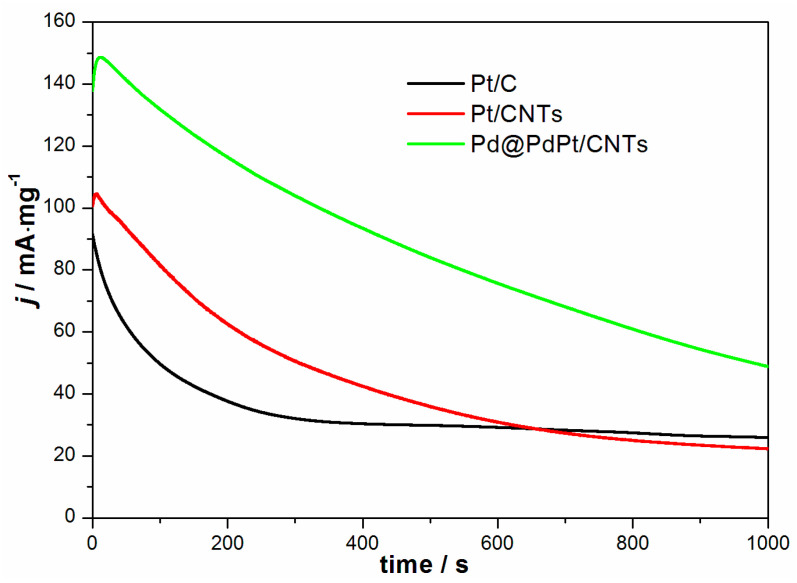
Chronoamperometric curves of different electrocatalysts in 0.1 M H_2_SO_4_ + 0.1 M methanol, 0.45 V vs. SCE.

Long term stability is a critical issue for developing efficient electrocatalysts. The chronoamperometry results of Pt/C, Pt/CNTs and Pd@PdPt/CNTs in 0.1 M H_2_SO_4_ + 0.1 M methanol are displayed in Figure 6. The polarization potential was set at 0.45 V and was held for 1000 s. The Pd@PdPt/CNTs exhibited the highest current response throughout the whole measurement. The retained current for Pd@PdPt/CNTs, Pt/C and Pt/CNTs were 33.4%, 28.3% and 21.2% respectively, demonstrating that the Pd@PdPt/CNT exhibits the highest stability among the three electrocatalysts. To further investigate the stability of the as-synthesized Pd@PdPt/CNTs electrocatalyst, the accelerated durability test (ADT) was also performed by conducting continued CV cycles. The as-synthesized Pd@PdPt/CNTs electrocatalyst achieves high stability for 10 CV cycles without a noticeable change.

## 4. Conclusions

Pd@PdPt/CNTs nanoparticles electrocatalyst was successfully synthesized through a two-step method. A surfactant-free monodispersed Pd/CNTs as a precursor was prepared by hydrolysis of [PdCl_4_]^2−^ in the presence of CNTs and subsequently by hydrogen reduction. The Pd@PdPt alloy core-shell electrocatalysts were obtained through the galvanic replacement reaction. The synthetic method is environmentally benign since no surfactants and capping agents are involved, demonstrating an effective and simple route for preparing monometallic or bimetallic nanoparticles with an absolutely clean surfactant-free electrocatalytic surface. It is quite promising for large-scale preparation. The Pd@PdPt/CNTs electrocatalyst exhibits a high electrochemical activity and excellent stability towards direct methanol oxidation.

## Figures and Tables

**Table 1:** pH value of the relevant solutions.

Solution	pH
Milli-Q water	5.62
CNTs + Milli-Q water	5.32
K_2_PdCl_4_	4.21
CNTs + K_2_PdCl_4_	3.45

**Table 2:** The Specific mass activity for Pd containing electrocatalysts towards methanol oxidation reaction.

Electrocatalyst	Specific Mass Activity (mAmg_Pt_^−1^)	Reference
PtPd nanocubes	340	[53]
PtCo nanoflowers	380	[54]
Pt/C	142.8	-
Pt/CNTs	164.1	-
Pd@PdPt/CNTs (This study)	400.2	-
